# Differential Gene Expression Analysis in a Lumbar Spinal Stenosis Rat Model via RNA Sequencing: Identification of Key Molecular Pathways and Therapeutic Insights

**DOI:** 10.3390/biomedicines13010192

**Published:** 2025-01-14

**Authors:** Jin Young Hong, Wan-Jin Jeon, Hyunseong Kim, Changhwan Yeo, Hyun Kim, Yoon Jae Lee, In-Hyuk Ha

**Affiliations:** Jaseng Spine and Joint Research Institute, Jaseng Medical Foundation, Seoul 135-896, Republic of Korea; vrt23@jaseng.org (J.Y.H.); cool2305@jaseng.org (W.-J.J.); biology@jaseng.org (H.K.); duelf2@gmail.com (C.Y.); khyeon94@jaseng.org (H.K.); goodsmile@jaseng.org (Y.J.L.)

**Keywords:** lumbar spinal stenosis, RNA sequencing, differential gene expression, molecular pathways, therapeutic targets

## Abstract

Background/Objectives: Lumbar spinal stenosis (LSS) is a degenerative condition characterized by the narrowing of the spinal canal, resulting in chronic pain and impaired mobility. However, the molecular mechanisms underlying LSS remain unclear. In this study, we performed RNA sequencing (RNA-seq) to investigate differential gene expression in a rat LSS model and identify the key genes and pathways involved in its pathogenesis. Methods: We used bioinformatics analysis to identify significant alterations in gene expression between the LSS-induced and sham groups. Results: Pearson’s correlation analysis demonstrated strongly consistent intragroup expression (r > 0.9), with distinct gene expression between the LSS and sham groups. A total of 113 differentially expressed genes (DEGs) were identified, including upregulated genes such as *Slc47a1* and *Prg4* and downregulated genes such as *Higd1c* and *Mln*. Functional enrichment analysis revealed that these DEGs included those involved in key biological processes, including synaptic plasticity, extracellular matrix organization, and hormonal regulation. Gene ontology analysis highlighted critical molecular functions such as mRNA binding and integrin binding, as well as cellular components such as contractile fibers and the extracellular matrix, which were significantly affected by LSS. Conclusions: Our findings provide novel insights into the molecular mechanisms underlying LSS and offer potential avenues for the development of targeted therapies aimed at mitigating disease progression and improving patient outcomes.

## 1. Introduction

Lumbar spinal stenosis (LSS) is a common degenerative condition that affects the older population and is characterized by the narrowing of the spinal canal, which leads to compression of the spinal nerves [1,2]. This condition results in a spectrum of symptoms, including chronic back pain, neurogenic claudication, and reduced mobility, significantly affecting the quality of life of the affected individual [2,3]. While LSS is often attributed to age-related degeneration of intervertebral discs, ligaments, and facet joints, the underlying molecular mechanisms driving these pathological changes remain poorly understood. Understanding these mechanisms is essential for developing novel and effective therapeutic strategies.

Current treatments for LSS primarily focus on symptom management, including physical therapy, anti-inflammatory medications, and surgical decompression in severe cases [4]. However, these interventions often fail to address the underlying molecular pathology of the disease, resulting in variable outcomes and high rates of symptom recurrence. Understanding the genetic and molecular pathways involved in LSS could provide critical insights into disease progression and help identify potential therapeutic targets that may halt or reverse its course.

Recent advances in transcriptomic technologies such as RNA sequencing (RNA-seq) have provided powerful tools for exploring global changes in gene expression associated with various diseases. RNA-seq offers high-resolution genome-wide insights into differentially expressed genes (DEGs) and their associated pathways, enabling researchers to identify key molecular players involved in disease processes [5,6]. In particular, RNA-seq can shed light on biological pathways and gene networks altered in degenerative diseases such as LSS, offering new avenues for therapeutic intervention. Additionally, although humans and rats have entirely different genomes, the rat model is widely used as a valuable tool to study the pathological processes of human diseases, because of shared physiological and genetic pathways [7,8,9]. This is particularly important in complex degenerative diseases, such as LSS, where direct gene analysis in humans is limited. Identifying specific genes and pathways in animal models is a critical first step in clinical research.

In this study, we performed RNA-seq to analyze the differential gene expression in a rat model of LSS, to elucidate the molecular alterations underlying the disease. By comparing the gene expression profiles of LSS-induced and sham rats, we aimed to identify the key genes and biological pathways involved in LSS pathogenesis. Additionally, we conducted bioinformatics analyses, including gene ontology (GO) enrichment, to investigate the functional roles of the identified DEGs. Our findings provide novel insights into the molecular landscape of LSS and identify potential therapeutic targets for mitigating disease progression and improving treatment outcomes.

## 2. Materials and Methods

### 2.1. Surgical Procedure for LSS Induction

All experimental procedures were reviewed and approved by the Jaseng Animal Care and Use Committee (IACUC No. JSR-2021-02-001-A). The manuscript was prepared according to the Animal Research: Reporting In Vivo Experiments (ARRIVE) guidelines. Male Sprague–Dawley (SD) rats, 7 weeks old and weighing 230–250 g, were acquired from Daehan BioLink. The surgical techniques were as described in detail in previous studies [10]. Briefly, animals were anesthetized with 2–3% isoflurane gas (Forane; BK Pham, Goyang, Korea), and dorsal laminectomy was performed at the L5 vertebral level using fine rongeurs. A silicone block (80 kPa, 4 mm × 1 mm × 1 mm) was carefully implanted at the L4 level using fine forceps. In the sham group, laminectomy was performed only at L5 without implantation. The animals were four male rats per group, sacrificed 4 weeks after LSS induction.

### 2.2. RNA Extraction and Quality Assessment

RNA was extracted from spinal cord tissue samples using the mirVana™ miRNA Isolation Kit (Ambion; Thermo Fisher Scientific, Waltham, MA, USA), following the manufacturer’s protocol. For each tissue sample, 10 volumes of Lysis/Binding Buffer relative to the tissue mass (600 μL of buffer) were aliquoted into a homogenization vessel on ice. The tissues were disrupted in cold Lysis/Binding Buffer using a TissueLyser (Qiagen, Hilden, Germany). Subsequently, 1/10 volume of the miRNA Homogenate Additive was added to the tissue lysate, and the mixture was vortexed or inverted several times to ensure thorough mixing before being left on ice for 10 min. An equal volume of acid phenol was added to the lysate and vortexed for 30–60 s. The mixture was then centrifuged at 10,000× *g* for 5 min. After phase separation, the supernatant was carefully transferred to a new tube, and 1.25 volumes of 100% ethanol (at room temperature) were added. The resulting lysate/ethanol mixture was applied to a filter cartridge inserted into a collection tube and centrifuged for approximately 15 s at 10,000× *g* to filter the mixture. This process was repeated until all mixtures passed through the filter.

To wash the RNA, 700 μL of Wash Solution 1 (prepared with ethanol) was added to the filter cartridge, followed by centrifugation for 5–10 s. The flow-through was discarded, and the process was repeated using Wash Solution 2/3 (prepared with ethanol) in two 500 μL aliquots. After the final wash, the filter cartridge was spun for 1 min to remove residual liquid. The filter cartridge was then transferred to a new collection tube, and 60 μL of pre-heated (95 °C) nuclease-free water was applied to the center of the filter to elute the RNA. After centrifugation for 20–30 s, the RNA was collected. RNA purity was assessed using a NanoDrop 2000 spectrophotometer (Thermo Fisher Scientific, Waltham, MA, USA), and RNA integrity was evaluated using an Agilent Bioanalyzer 2100 (Agilent Technologies, Santa Clara, CA, USA), with RNA integrity number values confirming the quality of the extracted RNA.

### 2.3. mRNA Library Preparation and Sequencing

mRNA sequencing libraries were prepared using a TruSeq Stranded mRNA Library Prep Kit (Illumina, San Diego, CA, USA) according to the manufacturer’s instructions. All experimental steps from library preparation to sequencing were performed by DNA Link Inc. (Seoul, Republic of Korea). mRNA was purified from the total RNA using poly-T oligo-attached magnetic beads and subjected to two rounds of purification. The purified mRNA was fragmented, and the cleaved RNA fragments were primed with random hexamers for reverse transcription into first-strand cDNA using reverse transcriptase. During this process, dUTP was substituted with dTTP. A single ‘A’ base was added to the cDNA fragments, followed by ligation of the adapters. The resulting products were purified and amplified using PCR to create a final strand-specific cDNA library. Library quality was confirmed using a tapestation system (Agilent Technologies, Santa Clara, CA, USA). Quantitative PCR (qPCR) was performed using KAPA SYBR FAST qPCR Master Mix (Kapa Biosystems, Wilmington, MA, USA) to ensure proper indexing, and libraries were pooled in equimolar amounts. Sequencing was then conducted on an Illumina NovaSeq 6000 platform following standard paired-end 2 × 100 bp sequencing protocols. To ensure the reliability of the sequencing results, quality control metrics were assessed, including raw reads, number of clean bases, and sequencing error rate. Detailed quality control metrics are provided in Supplementary Table S1.

### 2.4. Sequencing Data Processing and Normalization

Sequencing data were generated using paired-end (2 × 100 bp) reads on the Illumina NovaSeq 6000 platform, with 80% of the reads achieving a quality score above 30. Reads from each sample were pseudo-aligned to the reference genome (GRCh38) using Kallisto (v0.46.1), which provided transcript quantification and gene count data. A filtering process was applied to the gene list to retain genes with counts per million (cpm) of two or more in at least one replicate. Expression values were normalized using the trimmed mean of M-values method in the edgeR package (v3.32.1) in R (v4.0.5) to ensure accurate expression comparisons across samples. Waffle chart visualizations were generated in GraphPad Prism (v.8.0.1).

### 2.5. DEG Analysis

Two filtering steps were applied to identify DEGs between the sham and LSS groups. First, genes with low log cpm (logCPM) values were excluded from further analyses. Differential expression analysis was performed for the remaining genes. DEGs were selected based on two criteria: a minimum two-fold change in expression and a false discovery rate (FDR) < 0.05. This ensured that only genes with statistically significant and biologically meaningful changes in expression were considered for further interpretation.

### 2.6. GO and KEGG Pathway Enrichment Analysis

For the ontology analysis, genes that passed the previously mentioned thresholds were used as input for the DAVID tool to obtain a comprehensive set of functional annotations. Categories such as disease, GO, and pathways were selected for analysis. To broaden the output, the Ease score was adjusted from 0.1 to 1. The Ease score is a conservative modification of the Fisher’s exact test probability, emphasizing associations supported by more genes. DAVID generates a functional annotation chart that lists relevant annotation terms and their associated genes. In addition, GO and KEGG pathway enrichment analyses were performed using the “clusterProfiler” (v3.18.1) R package. GO terms and KEGG pathways with *p*-values < 0.05 were considered significant. GO functional enrichment analysis was conducted for biological processes (BPs), molecular functions (MFs), and cellular components (CCs). KEGG pathway visualizations were created using the “pathview” (v1.30.1) R package, and network graphs were generated using the “cnetplot” function of the “clusterProfiler” package.

## 3. Results

### 3.1. Correlation and Heatmap Analysis of Gene Expression in LSS and Sham Groups

**Figure 1:**
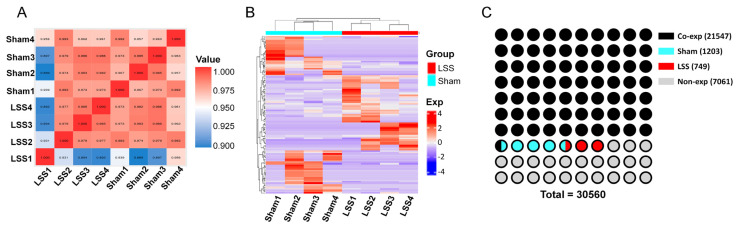
Whole-genome RNA sequencing analysis and gene expression patterns in sham and LSS groups. (**A**) Pearson’s correlation matrix (8 × 8) of gene expression profiles between the sham and LSS groups, showing correlation values between 0.88 and 1.0. (**B**) Heatmap of gene expression patterns clustered by gene type, illustrating distinct clustering between the sham and LSS groups. (**C**) Waffle chart summarizing RNA-seq results based on 30,560 rat reference genes.

To assess genome-wide expression patterns in the LSS model, we performed whole-genome RNA sequencing analyses. The Pearson’s correlation analysis was used to compare gene expression profiles between the sham and LSS groups. The resulting 8 × 8 symmetric matrices showed correlation values ranging from 0.88 to 1, indicating a high degree of similarity among the samples within each group (Figure 1A). Within the sham group, the samples exhibited consistently high correlation coefficients, with Sham1, Sham2, Sham3, and Sham4 showing average correlation coefficients of 0.967, 0.974, 0.970, and 0.974, respectively. In contrast, the LSS group samples, while still showing high correlations, displayed slightly lower coefficients than those in the sham group, with Sham1, Sham2, Sham3, and Sham4 having values of 0.966, 0.962, 0.957, and 0.967, respectively. Interestingly, the LSS1 sample in the LSS group exhibited a notably lower correlation range (0.889–0.956) than the other samples, suggesting that LSS1 had a unique gene expression pattern compared with the other LSS samples. To analyze the actual gene expression patterns, we performed clustering of expressed genes using a heatmap. The results showed that samples in the sham and LSS groups clustered distinctly according to gene types (Figure 1B). This indicated differences in gene expression patterns between the groups, suggesting that LSS induction led to changes in gene expression. To examine gene expression across the groups, we conducted RNA-seq analysis based on 30,560 reference genes derived from rats, identifying genes read in each group (Figure 1C). The RNA-seq results showed that 21,547 genes were read in the two groups, with 1203 genes expressed only in the sham group and 749 genes expressed only in the LSS group. Additionally, 7061 genes were not expressed in either group.

### 3.2. DEG Analysis and Identification of Key Genes in the LSS Model

**Figure 2:**
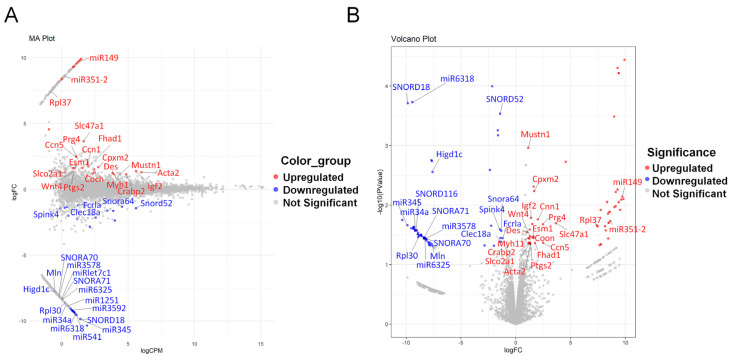
DEG analysis between sham and LSS groups. (**A**) MA plot showing DEG expression by plotting log fold change (logFC) against the average expression levels (logCPM) between sham and LSS groups. (**B**) Volcano plot illustrating the statistical significance (−log10 (*p*-value)) of expression changes between sham and LSS groups.

We identified differentially expressed genes between groups through RNA-seq analysis and filtered genes with a read count > 2 to identify those regulated by LSS induction. This process revealed a total of 15,465 genes, among which 59 genes were upregulated and 54 genes were downregulated (Supplementary Table S2). The MA plot illustrates the differential gene expression between the sham and LSS groups by visualizing the log fold change (logFC) against the average expression levels (logCPM) (Figure 2A). In this plot, red indicates upregulated genes, blue represents downregulated genes, and grey denotes statistically non-significant genes. Although most genes did not show statistically significant changes in expression, *Slc47a1* and *Prg4* were notable in the LSS group, displaying relatively high logCPM and a significant increase in logFC. This suggests that these genes play important roles in the pathophysiology of LSS. Conversely, *Acta2* and *Igf2* had some of the highest logCPM values; yet, they exhibited no significant changes in expression. This suggests that while these genes are highly expressed, they may not have a direct association with LSS. In contrast, *Higd1c* and *Mln* exhibited significant decreases in logFC and very low logCPM, indicating substantial reductions in their expression levels in the LSS group.

Furthermore, the volcano plot evaluating both expression changes and their statistical significance confirmed the prominent upregulation of *Slc47a1* and *Prg4* in the LSS group, with these genes showing high logFC and −log10 (*p*-values), reinforcing the statistical significance of their increased expression (Figure 2B). In contrast, *Higd1c* showed a marked decrease in expression, supported by a high −log10 (*p*-value), indicating significant downregulation. *Mln* displayed a similar trend but with a relatively lower −log10 (*p* value) than *Higd1c*. Genes such as *Mustn1* and *Cpxm2* had statistically significant *p*-values but exhibited relatively low logFC values, suggesting that their small expression changes, although statistically significant, may not have substantial biological implications. *Acta2* and *Igf2*, despite their high logCPM values, did not show statistically significant increases in expression, suggesting that these genes may not be directly related to LSS.

Overall, the MA and volcano plot analysis revealed that *Slc47a1* and *Prg4* were significantly upregulated and *Higd1c* was significantly downregulated in the LSS model. These findings provide important insights into the molecular mechanisms underlying LSS and may help identify novel therapeutic targets.

### 3.3. GO Enrichment of DEGs in LSS Model

**Figure 3:**
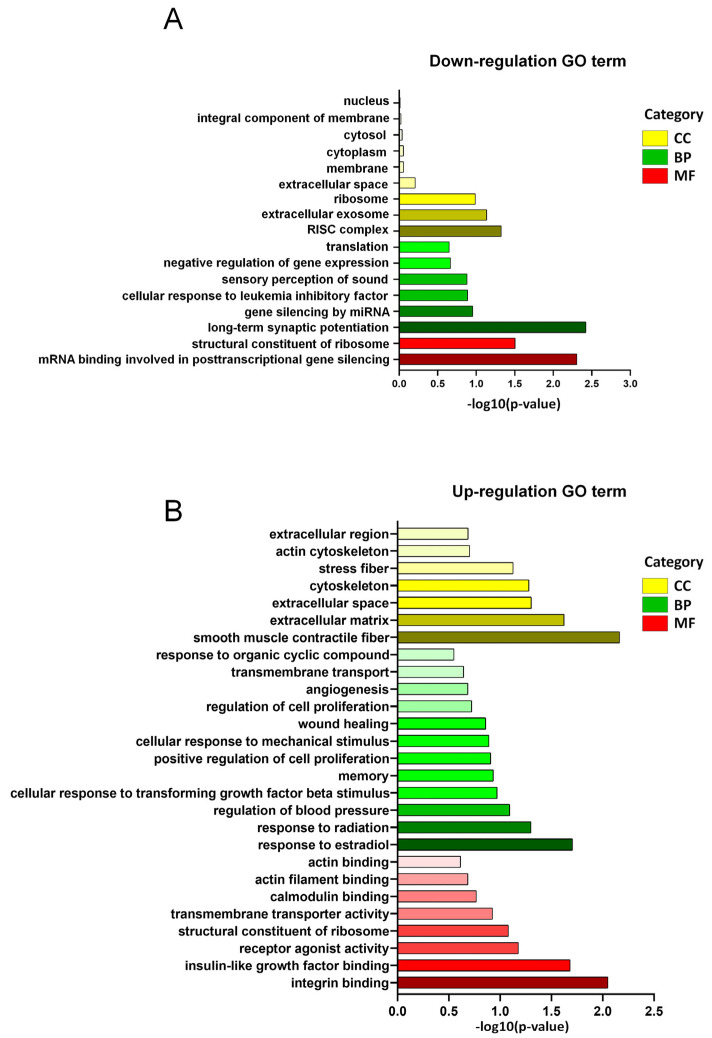
GO enrichment analysis of differentially expressed genes (DEGs) in the LSS model. (**A**) Downregulated GO terms in the LSS group compared with the sham group, displayed as a bar graph representing the statistical significance of each term’s downregulation (−log10 (*p*-value)). (**B**) Upregulated GO terms in the LSS group, identified through DEG analysis, with a total of 435 enriched terms including 58 MF-related, 262 BP-related, and 63 CC-related.

In our GO enrichment analysis of the LSS model, we investigated the association of DEGs with various biological processes (BPs), cellular components (CCs), and molecular functions (MFs). The bar graph in Figure 3A illustrates GO terms that were significantly downregulated in the LSS group compared with the sham group. The length of each bar represents the statistical significance of the enrichment of each GO term as −log10 (*p*-value). Our analysis of downregulated GO terms identified 104 GO terms where DEGs were significantly enriched, comprising 10 MF-related terms, 66 BP-related terms, and 22 CC-related terms (Supplementary Table S3). Among these, the most statistically significant biological process was ‘long-term synaptic potentiation’, with a −log10 (*p*-value) of 2.425. The second most significant molecular function-related GO term was ‘mRNA binding involved in posttranscriptional gene silencing’, with a −log10 (*p*-value) of 2.306. The third significant molecular function was ‘structural constituent of ribosome’, with a −log10 (*p*-value) of 1.505. Additionally, the CC-related ‘RISC complex’ with a −log10 (*p*-value) of 1.325 also showed significance. The significant reductions in these components suggest potential impacts on biological regulatory processes and neural function.

In the analysis of upregulated GO terms in the DEGs for the LSS model, a total of 435 GO terms were identified across MF, BP, and CC. Specifically, the analysis revealed 58 MF-related terms, 262 BP-related terms, and 63 CC-related terms, reflecting a broad spectrum of biological activities and pathways influenced by LSS (Supplementary Table S4).

Among the upregulated cellular components, ‘smooth muscle contractile fiber’ showed the highest statistical significance, with a log10 (*p*-value) of 2.164, indicating potential alterations in muscle contractility that could affect various physiological processes (Figure 3B). Another significantly enriched molecular function was ‘integrin binding’, reflecting changes in cellular adhesion and interactions with the extracellular matrix. Additionally, terms relating to ‘extracellular matrix’ and ‘extracellular space’ were also notably significant, suggesting structural changes in the extracellular environment that may impact tissue integrity and function.

In the biological processes category, the term ‘response to estradiol’ had a significant *p*-value, indicating that hormone-responsive pathways might be either a response to or a consequence of LSS. The significant changes observed in ‘insulin-like growth factor binding’ suggest possible alterations in growth factor signaling [11].

### 3.4. KEGG Enrichment Analysis of MF-Related Gene Expression Changes in the LSS Model

**Figure 4:**
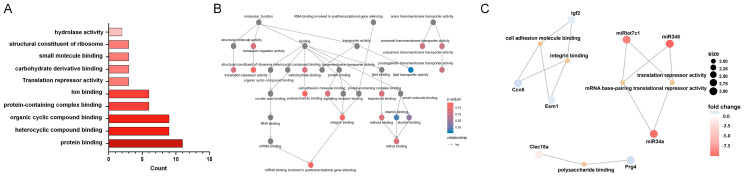
KEGG enrichment analysis of MF category in response to LSS induction. (**A**) Bar chart of KEGG pathway enrichment analysis of DEGs within the MF category. (**B**) Network showing connections between intersecting genes and various other MFs. (**C**) Network diagram visualizing interactions between specific genes and their MFs. Key genes include *Igf2*, *Ccn5*, *Esm1*, *Clec18a*, *Prg4*, and MicroRNA.

MF category analysis revealed that genes associated with protein binding exhibited the most substantial changes in response to LSS induction. This indicated that a significant number of genes involved in protein binding were affected by LSS, suggesting an adaptive cellular response to the altered mechanical environment (Figure 4A). Following protein binding, genes related to heterocyclic and organic cyclic compound binding showed considerable changes. Within the MF category, protein binding emerged as a central functional node connected to various other MFs. This suggests that protein binding plays a pivotal role in the coordination of diverse cellular and biological processes within the cell (Figure 4B). An additional network diagram visualizes the interactions between specific genes and their MFs (Figure 4C). *Igf2*, *Ccn5*, and *Esm1* are associated with cell adhesion molecules and integrin binding, essential for cell adhesion and signal transduction pathways. Additionally, *Clec18a* and *Prg4* are associated with the polysaccharide binding pathway, which contributes to the organization of the extracellular matrix and mediates interactions between cells and the extracellular matrix [12]. MicroRNAs such as miRlet7c1, miR345, and miR34a are linked to translation repression activity and primarily function by interacting with mRNA to regulate protein production.

### 3.5. KEGG Enrichment Analysis of BP-Related Gene Expression Changes in the LSS Model

**Figure 5:**
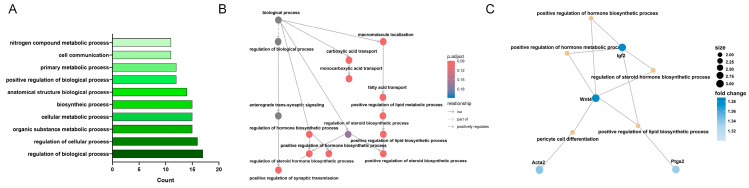
KEGG enrichment analysis of BP category in response to LSS induction. (**A**) Bar chart of KEGG pathway enrichment analysis of DEGs within the BP category. (**B**) Network showing connections between intersecting genes and various other BPs. (**C**) Network diagram visualizing interactions between specific genes and their BPs. Key genes include *Wnt4*, *Igf2*, and *Ptgs2*.

BP-related KEGG analysis indicated that many of the analyzed genes were involved in regulating BPs, suggesting that LSS induction significantly affected various biological pathways (Figure 5A).

The highlighted section of the network map presented in Figure 5B focuses on the most significant relationships and key BPs relevant to this study. For a comprehensive view, the complete network map illustrating all relationships and connections within the biological processes is available in Supplementary Figure S1. Among the BPs highlighted in this analysis, “regulation of biological process” is subdivided into multiple sub-processes, with some showing statistically significant changes in gene expression (Figure 5B). The positive regulation of synaptic transmission displayed notable changes, potentially enhancing neural functions associated with learning, memory, and responsiveness [13]. The observed gene changes related to hormone metabolism and cellular biosynthesis suggest upregulation of biosynthetic activity [14], which may support cell survival and function under LSS conditions [15]. Additionally, the regulation of steroid hormone biosynthetic processes ensures the proper synthesis of steroid hormones such as sex hormones. Significant changes in gene expression in this pathway suggest the potential modulation of steroid hormone levels, which could affect therapeutic outcomes, disease prevention, and general physiological activities. Furthermore, the positive regulation of hormone biosynthetic processes reflects the activation of genes that stimulate hormone production, which is essential for endocrine health and maintaining the function of various organs within the body [16]. This analysis is crucial for understanding how specific gene activation or suppression in various BPs affects disease progression and physiological states in LSS (Figure 5C). Among the genes showing notable changes in these BPs, *Wnt4* and *Igf2* are involved in the positive regulation of hormone biosynthetic processes, hormone metabolic processes, and steroid hormone biosynthetic processes. Additionally, *Wnt4* and *Ptgs2* are involved in the positive regulation of lipid biosynthetic processes, which could influence the hormonal balance between energy storage and utilization.

### 3.6. KEGG Enrichment Analysis of CC-Related Gene Expression Changes in the LSS Model

**Figure 6:**
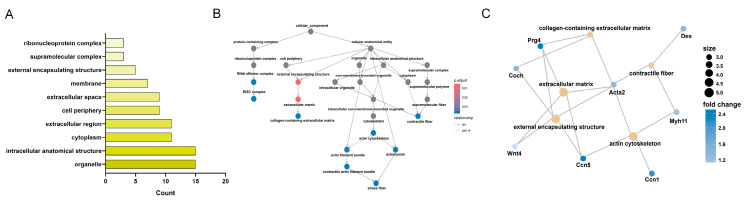
KEGG enrichment analysis of CC category in response to LSS induction. (**A**) Bar chart of KEGG pathway enrichment analysis of DEGs within the CC category. (**B**) Network showing connections between intersecting genes and various other CCs. (**C**) Network diagram visualizing interactions between specific genes and their BPs. Key genes include *Prg4*, *Ccn5*, *Coch*, *Wnt4*, *Acta2*, *Myh11*, and *Des*.

In the KEGG enrichment analysis in the CC category, changes in expression were observed in genes associated with organelles and intracellular anatomical structures, indicating that these cellular components were notably affected by LSS induction (Figure 6A). However, higher adjusted *p*-values were observed for genes related to external encapsulating structures and the extracellular matrix, implying that these components may play a substantial role in the response to LSS (Figure 6B). The network diagram highlights the specific genes linked to these CCs (Figure 6C). *Prg4*, *Ccn5*, *Coch*, *Wnt4*, and *Acta2* were strongly associated with intracellular and extracellular components. *Prg4* and *Ccn5* exhibited the highest expression levels. *Prg4*, together with *Acta2* and *Coch*, is associated with the collagen-containing extracellular matrix, emphasizing its role in maintaining extracellular structure. Meanwhile, *Ccn5* is involved in the actin cytoskeleton alongside *Acta2*, *Myh11*, and *Cnn1*, indicating its critical role in cytoskeletal stability and organization. Additionally, *Acta2* and *Myh11*, in association with *Des*, contribute to contractile fiber function, which is essential for cellular contractility. Network analysis suggested that while *Acta2* showed relatively lower expression than *Prg4* and *Ccn5*, it functioned as a crucial gene affecting multiple CCs, indicating its potential role as a key structural regulator within the cell. Furthermore, several genes, particularly *Igf2*, *Ccn5*, *Wnt4*, *Prg4*, and *Acta2*, exhibited significant changes in expression across various pathways, suggesting their potential as novel targets for understanding the genetic changes associated with LSS pathogenesis.

## 4. Discussion

In this study, we investigated changes in gene expression and pathway enrichment in an LSS rat model, to understand the molecular mechanisms underlying this condition. By analyzing DEGs, GO enrichment, and KEGG pathway involvement across various functional categories, we identified key genes and cellular processes that potentially contribute to the pathophysiology of LSS. Our findings highlighted significant alterations in cellular structure, signaling pathways, and metabolic regulation, suggesting novel therapeutic targets for LSS. Pearson’s correlation analysis and heatmap visualization revealed a high similarity in gene expression profiles within the sham and LSS groups. Although the sham and LSS groups generally displayed high correlation coefficients, the correlations between the two groups were slightly weaker than those within the sham group. This indicated the potential presence of subtle biological differences between the two groups, suggesting that distinct gene expression patterns existed despite the overall similarity. DEG analysis using MA and volcano plots identified *Slc47a1* and *Prg4* as significantly upregulated genes in the LSS model, while *Higd1c* showed substantial downregulation.

SLC47A1 is a multidrug and toxin extrusion transporter that plays an essential role in eliminating cationic substances, drugs, and toxins via the kidneys and liver, excreting them into the urine and bile, respectively [17,18]. SLC47A1 is also closely associated with the accumulation of anticancer drugs in cancer cells and can influence their efficacy [19]. For instance, high expression of SLC47A1 has been linked to reduced effectiveness of metformin in metastatic colorectal cancer and limited imatinib uptake in non-responding chronic myeloid leukemia patients. Furthermore, SLC47A1 is a significant predictor of the chemosensitivity of cancer cells to drugs, such as platinum–acridine compounds [19,20]. Recent studies have demonstrated that it suppresses ferroptosis through lipid remodeling in cells [21]. Despite these diverse roles, the specific function of SLC47A1 in the nervous system, particularly in LSS, remains unclear.

PRG4 is a mucin-like glycoprotein that plays critical roles in joint lubrication, synovial fluid homeostasis, and suppression of inflammation [22]. It binds to the CD44 receptor and exhibits anti-inflammatory, immunomodulatory, and anti-fibrotic effects [23,24,25]. In intervertebral disc-related research, PRG4 was abundantly expressed in the annulus fibrosus and identified as a nucleus pulposus-negative marker in healthy murine intervertebral discs [26]. Accordingly, *Prg4* has been suggested as a potential biomarker for degenerative disc diseases. In *Prg4*-knockout mouse model studies, PRG4 deficiency has been shown to increase the apparent torsional modulus of the disc and expand the nucleus pulposus area relative to the transverse disc area [27,28]. These findings suggest that PRG4 plays a critical role in maintaining the disc structure and function. *Higd1a* is a highly conserved gene expressed in various mammalian tissues [29]. HIGD1A is involved in key physiological processes in the nervous system, including neural proliferation, cell fate determination, and neuronal migration. It potentially contributes to cell preservation during programmed cell death, which occurs as the central nervous system matures [30]. In addition, HIGD1A has been associated with various diseases such as cancer, non-alcoholic fatty liver disease, type II diabetes, and mitochondrial diseases [29,31,32]; however, studies directly linking HIGD1A to LSS remain limited. These genes may play essential roles in LSS pathogenesis by contributing to cellular adaptation or maladaptation in response to LSS-induced mechanical stress. Notably, *Acta2* and *Igf2* demonstrated high expression levels without significant changes, suggesting that these genes, although abundantly expressed, may not be directly involved in the immediate response to LSS but could contribute to structural stability and basal cellular functions.

GO enrichment analysis highlighted significant changes in the BP, CC, and MF categories in the LSS model. The observed downregulation in “long-term synaptic potentiation” indicates potential impacts on neural plasticity, possibly affecting cognitive functions such as learning and memory [33]. This aligns with previous studies suggesting that disruptions in synaptic potentiation are linked to cognitive impairment in various neurological conditions [34,35]. Furthermore, the downregulation of ‘mRNA binding involved in post-transcriptional gene silencing’ indicates a potential reduction in post-transcriptional regulatory activity, which could disrupt normal gene-silencing processes. This disruption may lead to irregular patterns of gene expression, contributing to cellular dysfunction [36,37]. Additionally, the downregulation of functions related to the ‘structural constituent of ribosome’, which plays a crucial role in maintaining ribosome integrity for translating mRNA into amino acids [38], and the ‘RISC complex’, which is involved in RNA-induced post-transcriptional gene silencing using single-stranded RNA molecules like miRNA and siRNA to defend against double-stranded viral DNA infections [39,40], suggests possible impairments in gene regulation and neural function in the LSS model.

Among the upregulated terms, ‘smooth muscle contractile fibers’ and ‘integrin binding’ were notably enriched, indicating changes in cellular contractility and adhesion, which are essential for structural integrity and communication with the extracellular environment [41]. The term ‘smooth muscle contractile fiber’ is particularly relevant because smooth muscle fibers are critical for regulating blood pressure and blood flow in the vessels, airway modulation in the lungs, and motility and nutrient absorption in the gastrointestinal tract [42,43]. This suggests that LSS may influence smooth muscle function and potentially affect vital physiological processes.

Similarly, ‘integrin binding’ was also significantly upregulated. Integrins are transmembrane receptors that facilitate cell adhesion and play crucial roles in interactions with the extracellular matrix. This binding is fundamental for basal communication between cells and extracellular matrix components such as laminins, collagen, and fibronectin, indicating that changes in integrin activity can profoundly affect tissue architecture and disease pathology [41]. The significant enrichment of terms related to the ‘extracellular matrix’ and ‘extracellular space’ suggests that LSS influences the extracellular environment, potentially contributing to tissue remodeling or fibrosis, common in chronic spinal conditions [44,45].

Furthermore, changes in hormone-responsive pathways, such as the “response to estradiol” and “insulin-like growth factor binding”, underscore potential alterations in growth factor signaling pathways that are important for regulating growth hormones. This highlights the potential systemic impacts of LSS, possibly affecting growth factor signaling and hormonal regulation. These findings provide valuable insights into the complex molecular responses induced by LSS, with implications for targeting extracellular matrix regulation and hormonal pathways in therapeutic strategies. These hormonal interactions may have broad implications for cellular and organismal homeostasis [46].

The KEGG analysis results indicated notable changes in MFs associated with protein binding and interactions with organic compounds. This suggests that LSS induces extensive alterations in protein–protein interactions and signaling pathways. In the BP analysis, the regulation of genes associated with synaptic transmission, hormone metabolism, and biosynthesis was observed, indicating that LSS may support neural adaptation, cognitive function, and endocrine regulation. In terms of cellular structure, identified gene changes related to contractile fibers, the actin cytoskeleton, and stress fibers, suggesting that LSS affects intracellular structural stability and contractility. Additionally, genes such as *Prg4*, *Slc47a1*, *Igf2*, *Wnt4*, and *Acta2* play essential roles in cell adhesion, hormone regulation, and maintenance of extracellular matrix stability, suggesting their potential as therapeutic targets for tissue remodeling and mechanical stress adaptation under LSS conditions [12]. Notably, *Wnt4*, *Igf2*, and *Ptgs2* are involved in vascular support and regulation of lipid metabolism [47,48,49,50,51,52], which may contribute functionally to spinal disorders, whereas *Prg4* and *Ccn5* contribute to the extracellular matrix and cytoskeletal stability, supporting cellular structural adaptation induced by LSS. Although bioinformatics studies using spinal cord tissue for human LSS have not yet been reported, studies on hypertrophy of the ligamentum flavum, one of the primary causes of LSS, provide valuable insights. Other research has demonstrated that *Ccn5* exerts antifibrotic and antihypertrophic effects in human ligamentum flavum cells. Similarly, increased expression of *Prg4* has been observed in degenerated tissues, including ligaments surrounding affected areas [2]. These findings support the relevance of our results and suggest potential translational applications of the identified DEGs. Future studies integrating human ortholog data and validating the functional roles of these DEGs in clinical contexts would further enhance the understanding of LSS pathogenesis [53].

This study established a rat model of LSS by inserting a silicone block into the spinal canal to acutely induce stenosis, different from the gradual degenerative process through which human LSS develops. This difference presents a limitation in that the developed model does not fully replicate the natural process of disease progression in humans [54]. Moreover, the induction of stenosis through a single factor (silicone block insertion) differs from the multifactorial etiology of human LSS, which involves interactions among degeneration, genetic factors, and lifestyle factors. Therefore, this model may not fully capture the multifaceted nature of the disease. These differences could lead to variations in molecular pathways and gene expression profiles compared with those in humans. Additionally, because rats are quadrupedal animals, the mechanical loading and stress applied to the spine differ from those in humans. This may result in divergent biomechanical burdens and physiological responses to spinal stenosis, thereby limiting the direct applicability of the current study findings to humans. Furthermore, genomic differences between rats and humans may limit the generalizability of these study results to clinical applications.

Nonetheless, rat models remain valuable for exploring disease mechanisms and identifying potential therapeutic targets, because the primary molecular pathways relevant to disease onset often operate similarly in humans. Although this study successfully identified key genes and pathways associated with LSS through robust bioinformatic analyses, it is important to acknowledge the lack of experimental validation for these findings. The conclusions drawn are based exclusively on computational analyses, and further functional studies are essential to confirm the biological roles and mechanisms of the identified genes. Future research should focus on in vivo validation to provide deeper insights into the roles of these genes in tissue regeneration, inflammation, and fibrosis, which are critical processes in spinal degeneration. Such efforts would help bridge the gap between computational predictions and clinical applications, enhancing the translational value of these findings.

## 5. Conclusions

*Slc47a1* and *Prg4* were identified as key DEGs with upregulation, while *Higd1c* and *Mln* showed downregulation. GO and KEGG analyses revealed changes across various biological pathways, including synaptic potentiation, extracellular matrix remodeling, protein binding, and hormone metabolism. Notably, *Prg4* and *Acta2* are proposed as crucial biomarkers and therapeutic targets due to their significant roles in extracellular matrix organization and cytoskeletal stability, contributing to a deeper understanding of LSS pathophysiology and potential therapeutic development.

## Data Availability

The data presented in this study are available upon request from the corresponding author.

## Supplementary Materials

The following supporting information can be downloaded at: https://www.mdpi.com/article/10.3390/biomedicines13010192/s1, Table S1: Quality control metrics for RNA sequencing data; Table S2: Differentially expressed genes (DEGs) identified through RNA-seq analysis; Table S3: GO term analysis of downregulated genes; Table S4: GO term analysis of upregulated genes; Figure S1: Comprehensive network map of BP-related KEGG pathways.
