# Supplementary material for: Differential Gene Expression Analysis in a Lumbar Spinal Stenosis Rat Model via RNA Sequencing: Identification of Key Molecular Pathways and Therapeutic Insights
Source: Biomedicines. 2025 Jan 14;13(1):192. doi: 10.3390/biomedicines13010192 (PMC11762803; doi:10.3390/biomedicines13010192)
Supplement: Supplementary file 1 [file biomedicines-13-00192-s001.zip › biomedicines-3368842-supplementary.pdf]

Table S1. Quality control metrics for RNA sequencing data. The table provides detailed information on the quality of the sequencing data, including the number of raw reads, clean bases, and sequencing error rate for each sample.

Sample	Nanodrop Conc. (ng/㎕)	260/280	260/230	Total (㎍)	RIN	rRNA Ratio
Sham 1	586.8	1.91	2.17	19.36	6.9	0.7
Sham 2	84.9	2.03	1.91	2.80	7.6	0.9
Sham 3	100.5	2.02	1.14	3.31	7.3	0.8
Sham 4	105.5	1.95	1.63	3.48	7.9	1
LSS 1	66.3	1.93	1.54	2.19	7.1	1
LSS 2	146.3	2.00	1.66	4.83	7.8	1.1
LSS 3	267.9	1.99	2.01	8.84	8.3	1.4
LSS 4	223.2	2.01	0.85	7.37	8.2	1.6

Sample	Index	Reads	Yield (Mbases)	% of >= Q30 Bases (PF)	Mean QualityScore
Sham 1	ACTAAGAT+CCGCGGTT	51885614	5,240	93.78	36
Sham 2	GTCGGAGC+TTATAACC	56416188	5,698	96.04	36
Sham 3	CTTGGTAT+GGACTTGG	63771986	6,441	95.72	36
Sham 4	TCCAACGC+AAGTCCAA	54663586	5,521	95.84	36
LSS 1	CCGTGAAG+ATCCACTG	47230274	4,770	95.18	36
LSS 2	TTACAGGA+GCTTGTCA	49419282	4,991	94.18	36
LSS 3	GGCATTCT+CAAGCTAG	50331442	5,083	94.13	36
LSS 4	AATGCCTC+TGGATCGA	43463758	4,390	92.98	36

Table S2. Differentially expressed genes (DEGs) identified through RNA-seq analysis. The table lists genes filtered with a read count >2 and highlights those significantly regulated by LSS induction. It includes 59 upregulated genes and 54 downregulated genes, along with their corresponding fold changes, p-values, and functional annotations.

Comparison	Filtered_ID	UP	DOWN	TOTAL
1	15,465	59	54	113
* Filtered_ID : number of sample(read count > 2) in ID >= 1				
* UP(Case/Control) : p-value < 0.05 & log2FoldChange >= 1				
* DOWN(Case/Control) : p-value < 0.05 & log2FoldChange <= -1				

Table S3. GO term analysis of downregulated genes. The table provides a detailed list of 104 Gene Ontology (GO) terms significantly enriched by downregulated DEGs, categorized into Molecular Function (MF), Cellular Component (CC), and Biological Process (BP) terms. It includes 10 MF-related terms, 22 CC-related terms, and 66 BP-related terms, along with their enrichment scores and adjusted p-values.

Category	Term	p-value	Count
KEGG_PATHWAY	rno05206:MicroRNAs in cancer	0.0091808	3
KEGG_PATHWAY	rno03410:Base excision repair	1	1
KEGG_PATHWAY	rno05171:Coronavirus disease - COVID-19	1	1
KEGG_PATHWAY	rno04613:Neutrophil extracellular trap formation	1	1
KEGG_PATHWAY	rno04080:Neuroactive ligand-receptor interaction	1	1
KEGG_PATHWAY	rno04140:Autophagy - animal	1	1
KEGG_PATHWAY	rno03010:Ribosome	1	1
KEGG_PATHWAY	rno04217:Necroptosis	1	1
GOTERM_MF_DIRECT	GO:1903231~mRNA binding involved in posttranscriptional gene silencing	0.004939	3
GOTERM_MF_DIRECT	GO:0003735~structural constituent of ribosome	0.0312418	3
GOTERM_MF_DIRECT	GO:0004888~transmembrane signaling receptor activity	1	1
GOTERM_MF_DIRECT	GO:0031788~motilin receptor binding	1	1
GOTERM_MF_DIRECT	GO:0003730~mRNA 3'-UTR binding	1	1
GOTERM_MF_DIRECT	GO:0003677~DNA binding	1	1
GOTERM_MF_DIRECT	GO:0035368~selenocysteine insertion sequence binding	1	1
GOTERM_MF_DIRECT	GO:0003723~RNA binding	1	1
GOTERM_MF_DIRECT	GO:0004867~serine-type endopeptidase inhibitor activity	1	1
GOTERM_MF_DIRECT	GO:0030247~polysaccharide binding	1	1
GOTERM_CC_DIRECT	GO:0016442~RISC complex	0.0473368	2
GOTERM_CC_DIRECT	GO:0070062~extracellular exosome	0.0730919	2
GOTERM_CC_DIRECT	GO:0005840~ribosome	0.1027586	2
GOTERM_CC_DIRECT	GO:0005615~extracellular space	0.6210611	2
GOTERM_CC_DIRECT	GO:0016020~membrane	0.8780977	2
GOTERM_CC_DIRECT	GO:0005737~cytoplasm	0.8809651	3
GOTERM_CC_DIRECT	GO:0005829~cytosol	0.9135256	2
GOTERM_CC_DIRECT	GO:0016021~integral component of membrane	0.9495911	2
GOTERM_CC_DIRECT	GO:0005634~nucleus	0.9705312	2
GOTERM_CC_DIRECT	GO:0042788~polysomal ribosome	1	1
GOTERM_CC_DIRECT	GO:1990904~ribonucleoprotein complex	1	1
GOTERM_CC_DIRECT	GO:0005743~mitochondrial inner membrane	1	1
GOTERM_CC_DIRECT	GO:0045202~synapse	1	1
GOTERM_CC_DIRECT	GO:0005694~chromosome	1	1
GOTERM_CC_DIRECT	GO:0005887~integral component of plasma membrane	1	1
GOTERM_CC_DIRECT	GO:0005739~mitochondrion	1	1
GOTERM_CC_DIRECT	GO:0022627~cytosolic small ribosomal subunit	1	1
GOTERM_CC_DIRECT	GO:0014069~postsynaptic density	1	1
GOTERM_CC_DIRECT	GO:0022625~cytosolic large ribosomal subunit	1	1
GOTERM_CC_DIRECT	GO:0022626~cytosolic ribosome	1	1

GOTERM_BP_DIRECT	GO:0060291~long-term synaptic potentiation	0.003761	3
GOTERM_BP_DIRECT	GO:0035195~gene silencing by miRNA	0.110964	2
GOTERM_BP_DIRECT	GO:1990830~cellular response to leukemia inhibitory factor	0.1292521	2
GOTERM_BP_DIRECT	GO:0007605~sensory perception of sound	0.1317648	2
GOTERM_BP_DIRECT	GO:0010629~negative regulation of gene expression	0.2152097	2
GOTERM_BP_DIRECT	GO:0006412~translation	0.223865	2
GOTERM_BP_DIRECT	GO:0003323~type B pancreatic cell development	1	1
GOTERM_BP_DIRECT	GO:2000774~positive regulation of cellular senescence	1	1
GOTERM_BP_DIRECT	GO:0009410~response to xenobiotic stimulus	1	1
GOTERM_BP_DIRECT	GO:0051152~positive regulation of smooth muscle cell differentiation	1	1
GOTERM_BP_DIRECT	GO:0071356~cellular response to tumor necrosis factor	1	1
GOTERM_BP_DIRECT	GO:0031640~killing of cells of other organism	1	1
GOTERM_BP_DIRECT	GO:0010628~positive regulation of gene expression	1	1
GOTERM_BP_DIRECT	GO:0007566~embryo implantation	1	1
GOTERM_BP_DIRECT	GO:0035278~miRNA mediated inhibition of translation	1	1
GOTERM_BP_DIRECT	GO:1901985~positive regulation of protein acetylation	1	1
GOTERM_BP_DIRECT	GO:1904046~negative regulation of vascular endothelial growth factor production	1	1
GOTERM_BP_DIRECT	GO:0032717~negative regulation of interleukin-8 production	1	1
GOTERM_BP_DIRECT	GO:0097468~programmed cell death in response to reactive oxygen species	1	1
GOTERM_BP_DIRECT	GO:0061844~antimicrobial humoral immune response mediated by antimicrobial peptide	1	1
GOTERM_BP_DIRECT	GO:0097009~energy homeostasis	1	1
GOTERM_BP_DIRECT	GO:0016525~negative regulation of angiogenesis	1	1
GOTERM_BP_DIRECT	GO:0033209~tumor necrosis factor-mediated signaling pathway	1	1
GOTERM_BP_DIRECT	GO:0070482~response to oxygen levels	1	1
GOTERM_BP_DIRECT	GO:0006402~mRNA catabolic process	1	1
GOTERM_BP_DIRECT	GO:0071456~cellular response to hypoxia	1	1
GOTERM_BP_DIRECT	GO:1905563~negative regulation of vascular endothelial cell proliferation	1	1
GOTERM_BP_DIRECT	GO:0050829~defense response to Gram-negative bacterium	1	1
GOTERM_BP_DIRECT	GO:0007166~cell surface receptor signaling pathway	1	1
GOTERM_BP_DIRECT	GO:0032693~negative regulation of interleukin-10 production	1	1
GOTERM_BP_DIRECT	GO:0009617~response to bacterium	1	1
GOTERM_BP_DIRECT	GO:0071659~negative regulation of IP-10 production	1	1
GOTERM_BP_DIRECT	GO:0043524~negative regulation of neuron apoptotic process	1	1
GOTERM_BP_DIRECT	GO:0071230~cellular response to amino acid stimulus	1	1
GOTERM_BP_DIRECT	GO:0098868~bone growth	1	1
GOTERM_BP_DIRECT	GO:1904893~negative regulation of STAT cascade	1	1
GOTERM_BP_DIRECT	GO:0050859~negative regulation of B cell receptor signaling pathway	1	1
GOTERM_BP_DIRECT	GO:0034763~negative regulation of transmembrane transport	1	1
GOTERM_BP_DIRECT	GO:1904571~positive regulation of selenocysteine incorporation	1	1
GOTERM_BP_DIRECT	GO:0045087~innate immune response	1	1
GOTERM_BP_DIRECT	GO:0002181~cytoplasmic translation	1	1
GOTERM_BP_DIRECT	GO:0036035~osteoclast development	1	1
GOTERM_BP_DIRECT	GO:1903671~negative regulation of sprouting angiogenesis	1	1
GOTERM_BP_DIRECT	0035359~negative regulation of peroxisome proliferator activated receptor signaling path	1	1
GOTERM_BP_DIRECT	GO:2000108~positive regulation of leukocyte apoptotic process	1	1
GOTERM_BP_DIRECT	GO:0071222~cellular response to lipopolysaccharide	1	1
GOTERM_BP_DIRECT	GO:1902532~negative regulation of intracellular signal transduction	1	1
GOTERM_BP_DIRECT	GO:0032682~negative regulation of chemokine production	1	1
GOTERM_BP_DIRECT	GO:0006974~cellular response to DNA damage stimulus	1	1
GOTERM_BP_DIRECT	GO:1904706~negative regulation of vascular smooth muscle cell proliferation	1	1
GOTERM_BP_DIRECT	ndonucleolytic cleavage to generate mature 3'-end of SSU-rRNA from (SSU-rRNA, 5.8S rR	1	1
GOTERM_BP_DIRECT	GO:0071672~negative regulation of smooth muscle cell chemotaxis	1	1
GOTERM_BP_DIRECT	GO:2000738~positive regulation of stem cell differentiation	1	1
GOTERM_BP_DIRECT	GO:0030154~cell differentiation	1	1
GOTERM_BP_DIRECT	GO:0070104~negative regulation of interleukin-6-mediated signaling pathway	1	1
GOTERM_BP_DIRECT	GO:0061044~negative regulation of vascular wound healing	1	1
GOTERM_BP_DIRECT	GO:0045453~bone resorption	1	1
GOTERM_BP_DIRECT	GO:0001503~ossification	1	1
GOTERM_BP_DIRECT	GO:0010666~positive regulation of cardiac muscle cell apoptotic process	1	1
GOTERM_BP_DIRECT	GO:0097421~liver regeneration	1	1
GOTERM_BP_DIRECT	in ITS1 to separate SSU-rRNA from 5.8S rRNA and LSU-rRNA from tricistronic rRNA trans	1	1
GOTERM_BP_DIRECT	GO:0010884~positive regulation of lipid storage	1	1
GOTERM_BP_DIRECT	GO:0097250~mitochondrial respiratory chain supercomplex assembly	1	1
GOTERM_BP_DIRECT	GO:0042552~myelination	1	1
GOTERM_BP_DIRECT	GO:1904753~negative regulation of vascular associated smooth muscle cell migration	1	1
GOTERM_BP_DIRECT	GO:0090281~negative regulation of calcium ion import	1	1

Table S4. GO term analysis of upregulated genes. The table presents a comprehensive list of 435 Gene Ontology (GO) terms significantly enriched by upregulated DEGs in the LSS model. These terms are categorized into Molecular Function (MF, 58 terms), Biological Process (BP, 262 terms), and Cellular Component (CC, 63 terms), highlighting a wide range of biological activities and pathways influenced by LSS. Enrichment scores and adjusted p-values for each GO term are also provided.

Category	Term	p-value	Count
KEGG_PATHWAY	rno05200:Pathways in cancer	0.0908464	3
KEGG_PATHWAY	rno04270:Vascular smooth muscle contraction	0.125231	2
KEGG_PATHWAY	rno05225:Hepatocellular carcinoma	0.1604706	2
KEGG_PATHWAY	rno04814:Motor proteins	0.1711193	2
KEGG_PATHWAY	rno05205:Proteoglycans in cancer	0.1824527	2
KEGG_PATHWAY	rno03010:Ribosome	0.1968217	2
KEGG_PATHWAY	rno05171:Coronavirus disease - COVID-19	0.2668161	2
KEGG_PATHWAY	rno05165:Human papillomavirus infection	0.2926933	2
KEGG_PATHWAY	rno05010:Alzheimer disease	0.3198079	2
KEGG_PATHWAY	rno05022:Pathways of neurodegeneration - multiple diseases	0.3763595	2
KEGG_PATHWAY	rno04919:Thyroid hormone signaling pathway	1	1
KEGG_PATHWAY	rno04723:Retrograde endocannabinoid signaling	1	1
KEGG_PATHWAY	rno05410:Hypertrophic cardiomyopathy	1	1
KEGG_PATHWAY	rno04310:Wnt signaling pathway	1	1
KEGG_PATHWAY	rno05217:Basal cell carcinoma	1	1
KEGG_PATHWAY	rno04726:Serotonergic synapse	1	1
KEGG_PATHWAY	rno04010:MAPK signaling pathway	1	1
KEGG_PATHWAY	rno05163:Human cytomegalovirus infection	1	1
KEGG_PATHWAY	rno03040:Spliceosome	1	1
KEGG_PATHWAY	rno04926:Relaxin signaling pathway	1	1
KEGG_PATHWAY	rno04370:VEGF signaling pathway	1	1
KEGG_PATHWAY	rno04810:Regulation of actin cytoskeleton	1	1
KEGG_PATHWAY	rno01100:Metabolic pathways	1	1
KEGG_PATHWAY	rno04064:NF-kappa B signaling pathway	1	1
KEGG_PATHWAY	rno04014:Ras signaling pathway	1	1
KEGG_PATHWAY	rno04550:Signaling pathways regulating pluripotency of stem cells	1	1
KEGG_PATHWAY	rno05204:Chemical carcinogenesis - DNA adducts	1	1
KEGG_PATHWAY	rno05206:MicroRNAs in cancer	1	1
KEGG_PATHWAY	rno05226:Gastric cancer	1	1
KEGG_PATHWAY	rno04921:Oxytocin signaling pathway	1	1
KEGG_PATHWAY	rno04923:Regulation of lipolysis in adipocytes	1	1
KEGG_PATHWAY	rno04913:Ovarian steroidogenesis	1	1
KEGG_PATHWAY	rno04150:mTOR signaling pathway	1	1
KEGG_PATHWAY	rno04916:Melanogenesis	1	1
KEGG_PATHWAY	rno05414:Dilated cardiomyopathy	1	1
KEGG_PATHWAY	rno05224:Breast cancer	1	1
KEGG_PATHWAY	rno05222:Small cell lung cancer	1	1
KEGG_PATHWAY	rno04530:Tight junction	1	1
KEGG_PATHWAY	rno04371:Apelin signaling pathway	1	1
KEGG_PATHWAY	rno04668:TNF signaling pathway	1	1
KEGG_PATHWAY	rno04625:C-type lectin receptor signaling pathway	1	1
KEGG_PATHWAY	rno04148:Efferocytosis	1	1
KEGG_PATHWAY	rno05412:Arrhythmogenic right ventricular cardiomyopathy	1	1
KEGG_PATHWAY	rno03008:Ribosome biogenesis in eukaryotes	1	1
KEGG_PATHWAY	rno04657:IL-17 signaling pathway	1	1
KEGG_PATHWAY	rno05167:Kaposi sarcoma-associated herpesvirus infection	1	1
KEGG_PATHWAY	rno05140:Leishmaniasis	1	1
KEGG_PATHWAY	rno04390:Hippo signaling pathway	1	1
KEGG_PATHWAY	rno04360:Axon guidance	1	1
KEGG_PATHWAY	rno00590:Arachidonic acid metabolism	1	1
KEGG_PATHWAY	rno04934:Cushing syndrome	1	1
KEGG_PATHWAY	rno04151:PI3K-Akt signaling pathway	1	1

GOTERM_MF_DIRECT	GO:0005178~integrin binding	0.0088878	3
GOTERM_MF_DIRECT	GO:0005520~insulin-like growth factor binding	0.0207471	2
GOTERM_MF_DIRECT	GO:0048018~receptor agonist activity	0.0664027	2
GOTERM_MF_DIRECT	GO:0003735~structural constituent of ribosome	0.0829152	3
GOTERM_MF_DIRECT	GO:0022857~transmembrane transporter activity	0.1185451	2
GOTERM_MF_DIRECT	GO:0005516~calmodulin binding	0.1703374	2
GOTERM_MF_DIRECT	GO:0051015~actin filament binding	0.2055633	2
GOTERM_MF_DIRECT	GO:0003779~actin binding	0.2430504	2
GOTERM_MF_DIRECT	GO:0005515~protein binding	0.5070038	3
GOTERM_MF_DIRECT	GO:0005524~ATP binding	0.7675188	2
GOTERM_MF_DIRECT	GO:0046872~metal ion binding	0.8564388	2
GOTERM_MF_DIRECT	GO:0030247~polysaccharide binding	1	1
GOTERM_MF_DIRECT	GO:0005504~fatty acid binding	1	1
GOTERM_MF_DIRECT	GO:0015297~antiporter activity	1	1
GOTERM_MF_DIRECT	GO:0008307~structural constituent of muscle	1	1
GOTERM_MF_DIRECT	GO:0008201~heparin binding	1	1
GOTERM_MF_DIRECT	GO:0005125~cytokine activity	1	1
GOTERM_MF_DIRECT	GO:0019841~retinol binding	1	1
GOTERM_MF_DIRECT	GO:0005159~insulin-like growth factor receptor binding	1	1
GOTERM_MF_DIRECT	GO:0042803~protein homodimerization activity	1	1
GOTERM_MF_DIRECT	GO:0008270~zinc ion binding	1	1
GOTERM_MF_DIRECT	GO:0008083~growth factor activity	1	1
GOTERM_MF_DIRECT	GO:0016787~hydrolase activity	1	1
GOTERM_MF_DIRECT	GO:0008092~cytoskeletal protein binding	1	1
GOTERM_MF_DIRECT	GO:0005109~frizzled binding	1	1
GOTERM_MF_DIRECT	GO:0042887~amide transmembrane transporter activity	1	1
GOTERM_MF_DIRECT	GO:0004666~prostaglandin-endoperoxide synthase activity	1	1
GOTERM_MF_DIRECT	GO:0003714~transcription corepressor activity	1	1
GOTERM_MF_DIRECT	GO:0015489~putrescine transmembrane transporter activity	1	1
GOTERM_MF_DIRECT	GO:0019843~rRNA binding	1	1
GOTERM_MF_DIRECT	GO:0015347~sodium-independent organic anion transmembrane transporter activity	1	1
GOTERM_MF_DIRECT	GO:0019899~enzyme binding	1	1
GOTERM_MF_DIRECT	GO:0019901~protein kinase binding	1	1
GOTERM_MF_DIRECT	GO:0005044~scavenger receptor activity	1	1
GOTERM_MF_DIRECT	GO:0005518~collagen binding	1	1
GOTERM_MF_DIRECT	GO:0005158~insulin receptor binding	1	1
GOTERM_MF_DIRECT	ase activity, acting on single donors with incorporation of molecular oxygen, incorporatio	1	1
GOTERM_MF_DIRECT	GO:0005179~hormone activity	1	1
GOTERM_MF_DIRECT	GO:0030332~cyclin binding	1	1
GOTERM_MF_DIRECT	GO:0015101~organic cation transmembrane transporter activity	1	1
GOTERM_MF_DIRECT	GO:0004181~metallocarboxypeptidase activity	1	1
GOTERM_MF_DIRECT	GO:0015179~L-amino acid transmembrane transporter activity	1	1
GOTERM_MF_DIRECT	GO:0043539~protein serine/threonine kinase activator activity	1	1
GOTERM_MF_DIRECT	GO:0005171~hepatocyte growth factor receptor binding	1	1
GOTERM_MF_DIRECT	GO:0003774~motor activity	1	1
GOTERM_MF_DIRECT	GO:0001972~retinoic acid binding	1	1
GOTERM_MF_DIRECT	GO:0016918~retinal binding	1	1
GOTERM_MF_DIRECT	GO:0061459~L-arginine transmembrane transporter activity	1	1
GOTERM_MF_DIRECT	GO:0042910~xenobiotic transporter activity	1	1
GOTERM_MF_DIRECT	GO:0008289~lipid binding	1	1
GOTERM_MF_DIRECT	GO:0042802~identical protein binding	1	1
GOTERM_MF_DIRECT	GO:0051213~dioxygenase activity	1	1
GOTERM_MF_DIRECT	GO:0015234~thiamine transmembrane transporter activity	1	1
GOTERM_MF_DIRECT	GO:0003723~RNA binding	1	1
GOTERM_MF_DIRECT	GO:0004601~peroxidase activity	1	1
GOTERM_MF_DIRECT	GO:0030297~transmembrane receptor protein tyrosine kinase activator activity	1	1
GOTERM_MF_DIRECT	GO:0015132~prostaglandin transmembrane transporter activity	1	1
GOTERM_MF_DIRECT	GO:0020037~heme binding	1	1

GOTERM_CC_DIRECT	GO:0030485~smooth muscle contractile fiber	0.0068552	2
GOTERM_CC_DIRECT	GO:0031012~extracellular matrix	0.0237574	3
GOTERM_CC_DIRECT	GO:0005615~extracellular space	0.0495061	5
GOTERM_CC_DIRECT	GO:0005856~cytoskeleton	0.0523428	3
GOTERM_CC_DIRECT	GO:0001725~stress fiber	0.0746165	2
GOTERM_CC_DIRECT	GO:0015629~actin cytoskeleton	0.1979961	2
GOTERM_CC_DIRECT	GO:0005576~extracellular region	0.2048277	3
GOTERM_CC_DIRECT	GO:0005737~cytoplasm	0.3871618	7
GOTERM_CC_DIRECT	GO:0009986~cell surface	0.4803594	2
GOTERM_CC_DIRECT	GO:0032991~macromolecular complex	0.5502532	2
GOTERM_CC_DIRECT	GO:0005783~endoplasmic reticulum	0.6308831	2
GOTERM_CC_DIRECT	GO:0005634~nucleus	0.7264131	5
GOTERM_CC_DIRECT	GO:0016021~integral component of membrane	0.9377143	3
GOTERM_CC_DIRECT	GO:0005654~nucleoplasm	0.9469435	2
GOTERM_CC_DIRECT	GO:0005886~plasma membrane	0.9649757	3
GOTERM_CC_DIRECT	GO:0005829~cytosol	0.9772632	2
GOTERM_CC_DIRECT	GO:0005640~nuclear outer membrane	1	1
GOTERM_CC_DIRECT	GO:0005903~brush border	1	1
GOTERM_CC_DIRECT	GO:0030018~Z disc	1	1
GOTERM_CC_DIRECT	GO:0022626~cytosolic ribosome	1	1
GOTERM_CC_DIRECT	GO:0005859~muscle myosin complex	1	1
GOTERM_CC_DIRECT	GO:0045111~intermediate filament cytoskeleton	1	1
GOTERM_CC_DIRECT	GO:0031514~motile cilium	1	1
GOTERM_CC_DIRECT	GO:0005916~fascia adherens	1	1
GOTERM_CC_DIRECT	GO:0031982~vesicle	1	1
GOTERM_CC_DIRECT	GO:0005911~cell-cell junction	1	1
GOTERM_CC_DIRECT	GO:0022627~cytosolic small ribosomal subunit	1	1
GOTERM_CC_DIRECT	GO:0043292~contractile fiber	1	1
GOTERM_CC_DIRECT	GO:0030016~myofibril	1	1
GOTERM_CC_DIRECT	GO:0002102~podosome	1	1
GOTERM_CC_DIRECT	GO:0030175~filopodium	1	1
GOTERM_CC_DIRECT	GO:0045098~type III intermediate filament	1	1
GOTERM_CC_DIRECT	GO:0005869~dynactin complex	1	1
GOTERM_CC_DIRECT	GO:0014069~postsynaptic density	1	1
GOTERM_CC_DIRECT	GO:0014704~intercalated disc	1	1
GOTERM_CC_DIRECT	GO:0005844~polysome	1	1
GOTERM_CC_DIRECT	GO:0043005~neuron projection	1	1
GOTERM_CC_DIRECT	GO:0016020~membrane	1	1
GOTERM_CC_DIRECT	GO:0030027~lamellipodium	1	1
GOTERM_CC_DIRECT	GO:0005730~nucleolus	1	1
GOTERM_CC_DIRECT	GO:0005637~nuclear inner membrane	1	1
GOTERM_CC_DIRECT	GO:0042470~melanosome	1	1
GOTERM_CC_DIRECT	GO:0005794~Golgi apparatus	1	1
GOTERM_CC_DIRECT	GO:0005840~ribosome	1	1
GOTERM_CC_DIRECT	GO:0005604~basement membrane	1	1
GOTERM_CC_DIRECT	GO:0044297~cell body	1	1
GOTERM_CC_DIRECT	GO:0005789~endoplasmic reticulum membrane	1	1
GOTERM_CC_DIRECT	GO:0045202~synapse	1	1
GOTERM_CC_DIRECT	GO:0016324~apical plasma membrane	1	1
GOTERM_CC_DIRECT	GO:1990904~ribonucleoprotein complex	1	1
GOTERM_CC_DIRECT	GO:0005887~integral component of plasma membrane	1	1
GOTERM_CC_DIRECT	GO:0015935~small ribosomal subunit	1	1
GOTERM_CC_DIRECT	GO:0016459~myosin complex	1	1
GOTERM_CC_DIRECT	GO:0042383~sarcolemma	1	1
GOTERM_CC_DIRECT	GO:0005921~gap junction	1	1
GOTERM_CC_DIRECT	GO:0005764~lysosome	1	1
GOTERM_CC_DIRECT	GO:0005882~intermediate filament	1	1
GOTERM_CC_DIRECT	GO:0009925~basal plasma membrane	1	1
GOTERM_CC_DIRECT	GO:0032982~myosin filament	1	1
GOTERM_CC_DIRECT	GO:0005901~caveola	1	1
GOTERM_CC_DIRECT	GO:0031594~neuromuscular junction	1	1
GOTERM_CC_DIRECT	GO:0097512~cardiac myofibril	1	1
GOTERM_CC_DIRECT	GO:0016323~basolateral plasma membrane	1	1

GOTERM_BP_DIRECT	GO:0032355~response to estradiol	0.0196747	3
GOTERM_BP_DIRECT	GO:0009314~response to radiation	0.0500425	2
GOTERM_BP_DIRECT	GO:0008217~regulation of blood pressure	0.0805596	2
GOTERM_BP_DIRECT	GO:0071560~cellular response to transforming growth factor beta stimulus	0.1066087	2
GOTERM_BP_DIRECT	GO:0007613~memory	0.1163053	2
GOTERM_BP_DIRECT	GO:0008284~positive regulation of cell proliferation	0.1232924	3
GOTERM_BP_DIRECT	GO:0071260~cellular response to mechanical stimulus	0.1285022	2
GOTERM_BP_DIRECT	GO:0042060~wound healing	0.1379734	2
GOTERM_BP_DIRECT	GO:0042127~regulation of cell proliferation	0.1895592	2
GOTERM_BP_DIRECT	GO:0001525~angiogenesis	0.2055661	2
GOTERM_BP_DIRECT	GO:0055085~transmembrane transport	0.2267009	2
GOTERM_BP_DIRECT	GO:0014070~response to organic cyclic compound	0.2812083	2
GOTERM_BP_DIRECT	GO:0006412~translation	0.3545662	2
GOTERM_BP_DIRECT	GO:0008285~negative regulation of cell proliferation	0.3668272	2
GOTERM_BP_DIRECT	GO:0009410~response to xenobiotic stimulus	0.4278049	2
GOTERM_BP_DIRECT	GO:0010628~positive regulation of gene expression	0.466589	2
GOTERM_BP_DIRECT	GO:0007165~signal transduction	0.6196786	2
GOTERM_BP_DIRECT	GO:0031017~exocrine pancreas development	1	1
GOTERM_BP_DIRECT	GO:0045725~positive regulation of glycogen biosynthetic process	1	1
GOTERM_BP_DIRECT	GO:0045766~positive regulation of angiogenesis	1	1
GOTERM_BP_DIRECT	GO:0060548~negative regulation of cell death	1	1
GOTERM_BP_DIRECT	GO:1902475~L-alpha-amino acid transmembrane transport	1	1
GOTERM_BP_DIRECT	GO:0009615~response to virus	1	1
GOTERM_BP_DIRECT	GO:0001838~embryonic epithelial tube formation	1	1
GOTERM_BP_DIRECT	GO:0006955~immune response	1	1
GOTERM_BP_DIRECT	GO:0030326~embryonic limb morphogenesis	1	1
GOTERM_BP_DIRECT	GO:0014829~vascular smooth muscle contraction	1	1
GOTERM_BP_DIRECT	GO:0072033~renal vesicle formation	1	1
GOTERM_BP_DIRECT	GO:0006936~muscle contraction	1	1
GOTERM_BP_DIRECT	GO:0010033~response to organic substance	1	1
GOTERM_BP_DIRECT	GO:0098655~cation transmembrane transport	1	1
GOTERM_BP_DIRECT	GO:0040018~positive regulation of multicellular organism growth	1	1
GOTERM_BP_DIRECT	GO:0072144~glomerular mesangial cell development	1	1
GOTERM_BP_DIRECT	GO:0040037~negative regulation of fibroblast growth factor receptor signaling pathway	1	1
GOTERM_BP_DIRECT	GO:0035115~embryonic forelimb morphogenesis	1	1
GOTERM_BP_DIRECT	GO:0045165~cell fate commitment	1	1
GOTERM_BP_DIRECT	GO:0045786~negative regulation of cell cycle	1	1
GOTERM_BP_DIRECT	GO:1990961~drug transmembrane export	1	1
GOTERM_BP_DIRECT	GO:0030154~cell differentiation	1	1
GOTERM_BP_DIRECT	GO:0033080~immature T cell proliferation in thymus	1	1
GOTERM_BP_DIRECT	GO:0035567~non-canonical Wnt signaling pathway	1	1
GOTERM_BP_DIRECT	GO:0051897~positive regulation of protein kinase B signaling	1	1
GOTERM_BP_DIRECT	GO:0034198~cellular response to amino acid starvation	1	1
GOTERM_BP_DIRECT	GO:0051148~negative regulation of muscle cell differentiation	1	1
GOTERM_BP_DIRECT	GO:0090336~positive regulation of brown fat cell differentiation	1	1
GOTERM_BP_DIRECT	GO:2000225~negative regulation of testosterone biosynthetic process	1	1
GOTERM_BP_DIRECT	GO:0061045~negative regulation of wound healing	1	1
GOTERM_BP_DIRECT	GO:0010894~negative regulation of steroid biosynthetic process	1	1
GOTERM_BP_DIRECT	GO:0045840~positive regulation of mitotic nuclear division	1	1
GOTERM_BP_DIRECT	GO:0010042~response to manganese ion	1	1
GOTERM_BP_DIRECT	GO:0032227~negative regulation of synaptic transmission, dopaminergic	1	1
GOTERM_BP_DIRECT	GO:0051384~response to glucocorticoid	1	1

GOTERM_BP_DIRECT	GO:0008286~insulin receptor signaling pathway	1	1
GOTERM_BP_DIRECT	GO:0001889~liver development	1	1
GOTERM_BP_DIRECT	GO:1902730~positive regulation of proteoglycan biosynthetic process	1	1
GOTERM_BP_DIRECT	GO:0009887~animal organ morphogenesis	1	1
GOTERM_BP_DIRECT	GO:0009624~response to nematode	1	1
GOTERM_BP_DIRECT	GO:0072210~metanephric nephron development	1	1
GOTERM_BP_DIRECT	GO:2000066~positive regulation of cortisol biosynthetic process	1	1
GOTERM_BP_DIRECT	GO:0048009~insulin-like growth factor receptor signaling pathway	1	1
GOTERM_BP_DIRECT	GO:0001656~metanephros development	1	1
GOTERM_BP_DIRECT	GO:0015732~prostaglandin transport	1	1
GOTERM_BP_DIRECT	GO:0032332~positive regulation of chondrocyte differentiation	1	1
GOTERM_BP_DIRECT	GO:0045953~negative regulation of natural killer cell mediated cytotoxicity	1	1
GOTERM_BP_DIRECT	GO:0097190~apoptotic signaling pathway	1	1
GOTERM_BP_DIRECT	GO:0001503~ossification	1	1
GOTERM_BP_DIRECT	GO:0045944~positive regulation of transcription from RNA polymerase II promoter	1	1
GOTERM_BP_DIRECT	GO:0060231~mesenchymal to epithelial transition	1	1
GOTERM_BP_DIRECT	GO:0008543~fibroblast growth factor receptor signaling pathway	1	1
GOTERM_BP_DIRECT	GO:0010942~positive regulation of cell death	1	1
GOTERM_BP_DIRECT	GO:0001658~branching involved in ureteric bud morphogenesis	1	1
GOTERM_BP_DIRECT	GO:0071417~cellular response to organonitrogen compound	1	1
GOTERM_BP_DIRECT	GO:0009750~response to fructose	1	1
GOTERM_BP_DIRECT	GO:0034644~cellular response to UV	1	1
GOTERM_BP_DIRECT	GO:0072273~metanephric nephron morphogenesis	1	1
GOTERM_BP_DIRECT	GO:0008584~male gonad development	1	1
GOTERM_BP_DIRECT	GO:0006006~glucose metabolic process	1	1
GOTERM_BP_DIRECT	GO:0030325~adrenal gland development	1	1
GOTERM_BP_DIRECT	GO:0042445~hormone metabolic process	1	1
GOTERM_BP_DIRECT	GO:1905564~positive regulation of vascular endothelial cell proliferation	1	1
GOTERM_BP_DIRECT	GO:0072164~mesonephric tubule development	1	1
GOTERM_BP_DIRECT	GO:0071425~hematopoietic stem cell proliferation	1	1
GOTERM_BP_DIRECT	GO:0042274~ribosomal small subunit biogenesis	1	1
GOTERM_BP_DIRECT	GO:0019233~sensory perception of pain	1	1
GOTERM_BP_DIRECT	GO:0072034~renal vesicle induction	1	1
GOTERM_BP_DIRECT	GO:0035239~tube morphogenesis	1	1
GOTERM_BP_DIRECT	GO:0046697~decidualization	1	1
GOTERM_BP_DIRECT	GO:0007548~sex differentiation	1	1
GOTERM_BP_DIRECT	GO:0015908~fatty acid transport	1	1
GOTERM_BP_DIRECT	GO:0071464~cellular response to hydrostatic pressure	1	1
GOTERM_BP_DIRECT	GO:0045893~positive regulation of transcription, DNA-templated	1	1
GOTERM_BP_DIRECT	GO:0072174~metanephric tubule formation	1	1
GOTERM_BP_DIRECT	GO:0002040~sprouting angiogenesis	1	1
GOTERM_BP_DIRECT	GO:0060070~canonical Wnt signaling pathway	1	1
GOTERM_BP_DIRECT	GO:0001701~in utero embryonic development	1	1
GOTERM_BP_DIRECT	GO:0060008~Sertoli cell differentiation	1	1
GOTERM_BP_DIRECT	GO:0006855~drug transmembrane transport	1	1
GOTERM_BP_DIRECT	GO:1903826~arginine transmembrane transport	1	1
GOTERM_BP_DIRECT	GO:0030182~neuron differentiation	1	1
GOTERM_BP_DIRECT	GO:1990776~response to angiotensin	1	1
GOTERM_BP_DIRECT	GO:0060748~tertiary branching involved in mammary gland duct morphogenesis	1	1
GOTERM_BP_DIRECT	GO:2001234~negative regulation of apoptotic signaling pathway	1	1
GOTERM_BP_DIRECT	GO:2000491~positive regulation of hepatic stellate cell activation	1	1
GOTERM_BP_DIRECT	GO:0032967~positive regulation of collagen biosynthetic process	1	1
GOTERM_BP_DIRECT	GO:0071392~cellular response to estradiol stimulus	1	1
GOTERM_BP_DIRECT	GO:0030501~positive regulation of bone mineralization	1	1
GOTERM_BP_DIRECT	GO:0007155~cell adhesion	1	1
GOTERM_BP_DIRECT	GO:0045089~positive regulation of innate immune response	1	1

GOTERM_BP_DIRECT	GO:0051145~smooth muscle cell differentiation	1	1
GOTERM_BP_DIRECT	GO:0071456~cellular response to hypoxia	1	1
GOTERM_BP_DIRECT	GO:2000180~negative regulation of androgen biosynthetic process	1	1
GOTERM_BP_DIRECT	GO:0033138~positive regulation of peptidyl-serine phosphorylation	1	1
GOTERM_BP_DIRECT	GO:0042104~positive regulation of activated T cell proliferation	1	1
GOTERM_BP_DIRECT	GO:1902600~hydrogen ion transmembrane transport	1	1
GOTERM_BP_DIRECT	GO:0022407~regulation of cell-cell adhesion	1	1
GOTERM_BP_DIRECT	GO:0045836~positive regulation of meiotic nuclear division	1	1
GOTERM_BP_DIRECT	GO:1904238~pericyte cell differentiation	1	1
GOTERM_BP_DIRECT	GO:0002138~retinoic acid biosynthetic process	1	1
GOTERM_BP_DIRECT	GO:0032502~developmental process	1	1
GOTERM_BP_DIRECT	GO:0042573~retinoic acid metabolic process	1	1
GOTERM_BP_DIRECT	GO:0008360~regulation of cell shape	1	1
GOTERM_BP_DIRECT	GO:0031667~response to nutrient levels	1	1
GOTERM_BP_DIRECT	GO:0001823~mesonephros development	1	1
GOTERM_BP_DIRECT	GO:0097638~L-arginine import across plasma membrane	1	1
GOTERM_BP_DIRECT	GO:0001822~kidney development	1	1
GOTERM_BP_DIRECT	GO:0060720~spongiotrophoblast cell proliferation	1	1
GOTERM_BP_DIRECT	GO:0061180~mammary gland epithelium development	1	1
GOTERM_BP_DIRECT	GO:0010243~response to organonitrogen compound	1	1
GOTERM_BP_DIRECT	GO:0045109~intermediate filament organization	1	1
GOTERM_BP_DIRECT	GO:0006897~endocytosis	1	1
GOTERM_BP_DIRECT	GO:0051147~regulation of muscle cell differentiation	1	1
GOTERM_BP_DIRECT	GO:0043547~positive regulation of GTPase activity	1	1
GOTERM_BP_DIRECT	GO:0007566~embryo implantation	1	1
GOTERM_BP_DIRECT	GO:0006364~rRNA processing	1	1
GOTERM_BP_DIRECT	GO:0007568~aging	1	1
GOTERM_BP_DIRECT	GO:0030282~bone mineralization	1	1
GOTERM_BP_DIRECT	GO:0071318~cellular response to ATP	1	1
GOTERM_BP_DIRECT	GO:0008585~female gonad development	1	1
GOTERM_BP_DIRECT	GO:0015695~organic cation transport	1	1
GOTERM_BP_DIRECT	GO:0001516~prostaglandin biosynthetic process	1	1
GOTERM_BP_DIRECT	GO:0001934~positive regulation of protein phosphorylation	1	1
GOTERM_BP_DIRECT	GO:0089718~amino acid import across plasma membrane	1	1
GOTERM_BP_DIRECT	GO:0016485~protein processing	1	1
GOTERM_BP_DIRECT	GO:0042246~tissue regeneration	1	1
GOTERM_BP_DIRECT	GO:0032715~negative regulation of interleukin-6 production	1	1
GOTERM_BP_DIRECT	GO:0015847~putrescine transport	1	1
GOTERM_BP_DIRECT	GO:0045596~negative regulation of cell differentiation	1	1
GOTERM_BP_DIRECT	GO:0070374~positive regulation of ERK1 and ERK2 cascade	1	1
GOTERM_BP_DIRECT	GO:0071471~cellular response to non-ionic osmotic stress	1	1
GOTERM_BP_DIRECT	GO:0042742~defense response to bacterium	1	1
GOTERM_BP_DIRECT	GO:0051968~positive regulation of synaptic transmission, glutamatergic	1	1
GOTERM_BP_DIRECT	GO:0050731~positive regulation of peptidyl-tyrosine phosphorylation	1	1
GOTERM_BP_DIRECT	GO:0033280~response to vitamin D	1	1
GOTERM_BP_DIRECT	GO:0031056~regulation of histone modification	1	1
GOTERM_BP_DIRECT	GO:0032496~response to lipopolysaccharide	1	1
GOTERM_BP_DIRECT	GO:0006979~response to oxidative stress	1	1
GOTERM_BP_DIRECT	GO:0071498~cellular response to fluid shear stress	1	1
GOTERM_BP_DIRECT	GO:0001558~regulation of cell growth	1	1
GOTERM_BP_DIRECT	GO:0010226~response to lithium ion	1	1
GOTERM_BP_DIRECT	GO:0061041~regulation of wound healing	1	1
GOTERM_BP_DIRECT	GO:0061369~negative regulation of testicular blood vessel morphogenesis	1	1
GOTERM_BP_DIRECT	GO:0034605~cellular response to heat	1	1
GOTERM_BP_DIRECT	GO:0034612~response to tumor necrosis factor	1	1
GOTERM_BP_DIRECT	GO:0060669~embryonic placenta morphogenesis	1	1
GOTERM_BP_DIRECT	GO:0006939~smooth muscle contraction	1	1
GOTERM_BP_DIRECT	GO:0045907~positive regulation of vasoconstriction	1	1
GOTERM_BP_DIRECT	43154~negative regulation of cysteine-type endopeptidase activity involved in apoptotic p	1	1
GOTERM_BP_DIRECT	GO:0051781~positive regulation of cell division	1	1
GOTERM_BP_DIRECT	GO:0009617~response to bacterium	1	1
GOTERM_BP_DIRECT	GO:0043536~positive regulation of blood vessel endothelial cell migration	1	1
GOTERM_BP_DIRECT	GO:0006869~lipid transport	1	1
GOTERM_BP_DIRECT	GO:0051894~positive regulation of focal adhesion assembly	1	1
GOTERM_BP_DIRECT	GO:0051926~negative regulation of calcium ion transport	1	1
GOTERM_BP_DIRECT	GO:0055013~cardiac muscle cell development	1	1
GOTERM_BP_DIRECT	GO:0045471~response to ethanol	1	1
GOTERM_BP_DIRECT	GO:0060129~thyroid-stimulating hormone-secreting cell differentiation	1	1

GOTERM_BP_DIRECT	GO:0000028~ribosomal small subunit assembly	1	1
GOTERM_BP_DIRECT	GO:0045669~positive regulation of osteoblast differentiation	1	1
GOTERM_BP_DIRECT	GO:0042307~positive regulation of protein import into nucleus	1	1
GOTERM_BP_DIRECT	GO:0060993~kidney morphogenesis	1	1
GOTERM_BP_DIRECT	GO:0030237~female sex determination	1	1
GOTERM_BP_DIRECT	GO:0090362~positive regulation of platelet-derived growth factor production	1	1
GOTERM_BP_DIRECT	GO:0048672~positive regulation of collateral sprouting	1	1
GOTERM_BP_DIRECT	GO:0033077~T cell differentiation in thymus	1	1
GOTERM_BP_DIRECT	GO:0048856~anatomical structure development	1	1
GOTERM_BP_DIRECT	GO:0050873~brown fat cell differentiation	1	1
GOTERM_BP_DIRECT	GO:0090050~positive regulation of cell migration involved in sprouting angiogenesis	1	1
GOTERM_BP_DIRECT	GO:0048633~positive regulation of skeletal muscle tissue growth	1	1
GOTERM_BP_DIRECT	GO:0061184~positive regulation of dermatome development	1	1
GOTERM_BP_DIRECT	GO:0098869~cellular oxidant detoxification	1	1
GOTERM_BP_DIRECT	GO:0030728~ovulation	1	1
GOTERM_BP_DIRECT	GO:0032349~positive regulation of aldosterone biosynthetic process	1	1
GOTERM_BP_DIRECT	GO:0043252~sodium-independent organic anion transport	1	1
GOTERM_BP_DIRECT	GO:0031032~actomyosin structure organization	1	1
GOTERM_BP_DIRECT	GO:0090031~positive regulation of steroid hormone biosynthetic process	1	1
GOTERM_BP_DIRECT	GO:0048599~oocyte development	1	1
GOTERM_BP_DIRECT	GO:0010575~positive regulation of vascular endothelial growth factor production	1	1
GOTERM_BP_DIRECT	GO:0042633~hair cycle	1	1
GOTERM_BP_DIRECT	GO:0035633~maintenance of permeability of blood-brain barrier	1	1
GOTERM_BP_DIRECT	GO:0006518~peptide metabolic process	1	1
GOTERM_BP_DIRECT	GO:0048251~elastic fiber assembly	1	1
GOTERM_BP_DIRECT	GO:0031394~positive regulation of prostaglandin biosynthetic process	1	1
GOTERM_BP_DIRECT	GO:0046628~positive regulation of insulin receptor signaling pathway	1	1
GOTERM_BP_DIRECT	02219~negative regulation of intrinsic apoptotic signaling pathway in response to osmoti	1	1
GOTERM_BP_DIRECT	GO:0032526~response to retinoic acid	1	1
GOTERM_BP_DIRECT	GO:0006811~ion transport	1	1
GOTERM_BP_DIRECT	GO:0042908~xenobiotic transport	1	1
GOTERM_BP_DIRECT	GO:0038030~non-canonical Wnt signaling pathway via MAPK cascade	1	1
GOTERM_BP_DIRECT	GO:0001892~embryonic placenta development	1	1
GOTERM_BP_DIRECT	GO:0035988~chondrocyte proliferation	1	1
GOTERM_BP_DIRECT	GO:0051146~striated muscle cell differentiation	1	1

GOTERM_BP_DIRECT	GO:0060538~skeletal muscle organ development	1	1
GOTERM_BP_DIRECT	GO:0045986~negative regulation of smooth muscle contraction	1	1
GOTERM_BP_DIRECT	GO:0060126~somatotropin secreting cell differentiation	1	1
GOTERM_BP_DIRECT	GO:0007276~gamete generation	1	1
GOTERM_BP_DIRECT	GO:1904706~negative regulation of vascular smooth muscle cell proliferation	1	1
GOTERM_BP_DIRECT	GO:0009267~cellular response to starvation	1	1
GOTERM_BP_DIRECT	GO:0042886~amide transport	1	1
GOTERM_BP_DIRECT	GO:2000019~negative regulation of male gonad development	1	1
GOTERM_BP_DIRECT	GO:0045987~positive regulation of smooth muscle contraction	1	1
GOTERM_BP_DIRECT	GO:0061205~paramesonephric duct development	1	1
GOTERM_BP_DIRECT	GO:1902732~positive regulation of chondrocyte proliferation	1	1
GOTERM_BP_DIRECT	GO:0043065~positive regulation of apoptotic process	1	1
GOTERM_BP_DIRECT	GO:0001649~osteoblast differentiation	1	1
GOTERM_BP_DIRECT	GO:0007015~actin filament organization	1	1
GOTERM_BP_DIRECT	GO:0007612~learning	1	1
GOTERM_BP_DIRECT	GO:0045892~negative regulation of transcription, DNA-templated	1	1
GOTERM_BP_DIRECT	GO:0031915~positive regulation of synaptic plasticity	1	1
GOTERM_BP_DIRECT	GO:0032310~prostaglandin secretion	1	1
GOTERM_BP_DIRECT	GO:0006812~cation transport	1	1
GOTERM_BP_DIRECT	GO:0072006~nephron development	1	1

GOTERM_BP_DIRECT	GO:0035094~response to nicotine	1	1
GOTERM_BP_DIRECT	GO:0072051~juxtaglomerular apparatus development	1	1
GOTERM_BP_DIRECT	GO:0002062~chondrocyte differentiation	1	1
GOTERM_BP_DIRECT	GO:0045429~positive regulation of nitric oxide biosynthetic process	1	1
GOTERM_BP_DIRECT	GO:0043066~negative regulation of apoptotic process	1	1
GOTERM_BP_DIRECT	GO:0090271~positive regulation of fibroblast growth factor production	1	1
GOTERM_BP_DIRECT	GO:0007565~female pregnancy	1	1
GOTERM_BP_DIRECT	GO:0007605~sensory perception of sound	1	1
GOTERM_BP_DIRECT	GO:0034097~response to cytokine	1	1
GOTERM_BP_DIRECT	GO:0006954~inflammatory response	1	1
GOTERM_BP_DIRECT	GO:0019371~cyclooxygenase pathway	1	1
GOTERM_BP_DIRECT	GO:0071284~cellular response to lead ion	1	1
GOTERM_BP_DIRECT	GO:0030336~negative regulation of cell migration	1	1
GOTERM_BP_DIRECT	GO:0043085~positive regulation of catalytic activity	1	1
GOTERM_BP_DIRECT	GO:0043410~positive regulation of MAPK cascade	1	1
GOTERM_BP_DIRECT	GO:0090131~mesenchyme migration	1	1
GOTERM_BP_DIRECT	GO:2000467~positive regulation of glycogen (starch) synthase activity	1	1
GOTERM_BP_DIRECT	GO:0071636~positive regulation of transforming growth factor beta production	1	1
GOTERM_BP_DIRECT	GO:0010629~negative regulation of gene expression	1	1
GOTERM_BP_DIRECT	GO:0046622~positive regulation of organ growth	1	1
GOTERM_BP_DIRECT	GO:0048754~branching morphogenesis of an epithelial tube	1	1
GOTERM_BP_DIRECT	GO:0000122~negative regulation of transcription from RNA polymerase II promoter	1	1
GOTERM_BP_DIRECT	GO:0070542~response to fatty acid	1	1
GOTERM_BP_DIRECT	GO:0080021~response to benzoic acid	1	1
GOTERM_BP_DIRECT	GO:0001837~epithelial to mesenchymal transition	1	1
GOTERM_BP_DIRECT	GO:0071934~thiamine transmembrane transport	1	1
GOTERM_BP_DIRECT	GO:0038028~insulin receptor signaling pathway via phosphatidylinositol 3-kinase	1	1
GOTERM_BP_DIRECT	GO:0031622~positive regulation of fever generation	1	1
GOTERM_BP_DIRECT	GO:0048661~positive regulation of smooth muscle cell proliferation	1	1
GOTERM_BP_DIRECT	GO:0051496~positive regulation of stress fiber assembly	1	1
GOTERM_BP_DIRECT	GO:1902204~positive regulation of hepatocyte growth factor receptor signaling pathway	1	1

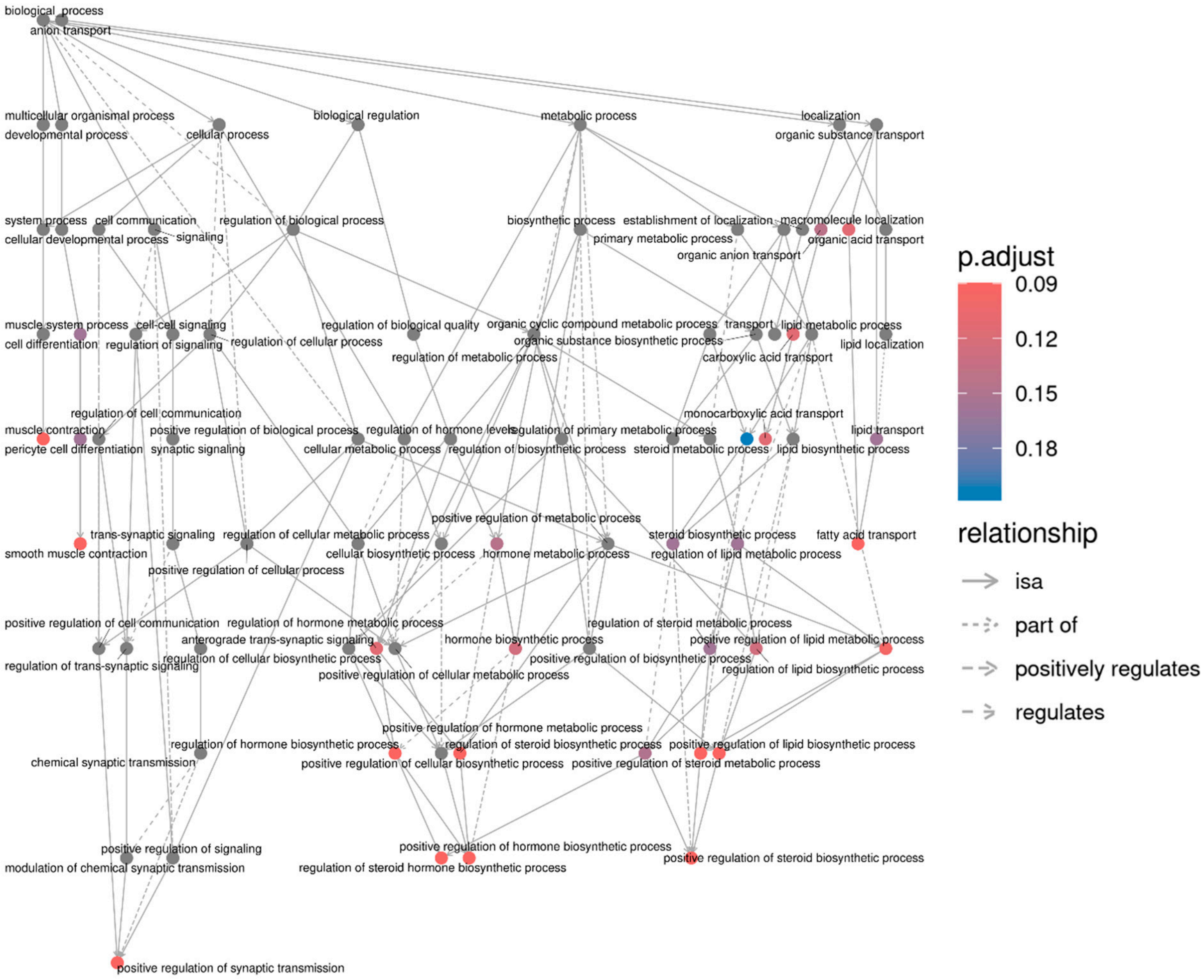

Figure S1. Comprehensive network map of BP-related KEGG pathways. This figure provides a complete overview of the network map illustrating all relationships and connections among BPs affected by LSS induction.
